# Sequestration of multiple RNA recognition motif-containing proteins by *C9orf72* repeat expansions

**DOI:** 10.1093/brain/awu120

**Published:** 2014-05-27

**Authors:** Johnathan Cooper-Knock, Matthew J. Walsh, Adrian Higginbottom, J. Robin Highley, Mark J. Dickman, Dieter Edbauer, Paul G. Ince, Stephen B. Wharton, Stuart A. Wilson, Janine Kirby, Guillaume M. Hautbergue, Pamela J. Shaw

**Affiliations:** 1 Sheffield Institute for Translational Neuroscience (SITraN), University of Sheffield, 385A Glossop Road, Sheffield S10 2HQ, UK; 2 Chemical and Biological Engineering, ChELSI Institute, University of Sheffield, Mappin Street, Sheffield, S1 3JD, UK; 3 DZNE–German Centre for Neurodegenerative Diseases and Munich Cluster of Systems Neurology (SyNergy), Munich, Germany; 4 Department of Molecular Biology and Biotechnology, University of Sheffield, Firth Court, Sheffield, S10 2TN, UK

**Keywords:** amyotrophic lateral sclerosis, pathology, genetics, fluorescence imaging

## Abstract

GGGGCC repeat expansions of *C9orf72* represent the most common genetic variant of amyotrophic lateral sclerosis and frontotemporal degeneration, but the mechanism of pathogenesis is unclear. Recent reports have suggested that the transcribed repeat might form toxic RNA foci that sequester various RNA processing proteins. Consensus as to the identity of the binding partners is missing and whole neuronal proteome investigation is needed. Using RNA fluorescence *in situ* hybridization we first identified nuclear and cytoplasmic RNA foci in peripheral and central nervous system biosamples from patients with amyotrophic lateral sclerosis with a repeat expansion of *C9orf72* (*C9orf72*+), but not from those patients without a repeat expansion of *C9orf72* (*C9orf72−*) or control subjects. Moreover, in the cases examined, the distribution of foci-positive neurons correlated with the clinical phenotype (*t*-test *P < *0.05). As expected, RNA foci are ablated by RNase treatment. Interestingly, we identified foci in fibroblasts from an asymptomatic *C9orf72*+ carrier. We next performed pulldown assays, with GGGGCC_5_, in conjunction with mass spectrometry analysis, to identify candidate binding partners of the GGGGCC repeat expansion. Proteins containing RNA recognition motifs and involved in splicing, messenger RNA nuclear export and/or translation were significantly enriched. Immunohistochemistry in central nervous system tissue from *C9orf72*+ patients with amyotrophic lateral sclerosis demonstrated co-localization of RNA foci with SRSF2, hnRNP H1/F, ALYREF and hnRNP A1 in cerebellar granule cells and with SRSF2, hnRNP H1/F and ALYREF in motor neurons, the primary target of pathology in amyotrophic lateral sclerosis. Direct binding of proteins to GGGGCC repeat RNA was confirmed *in vitro* by ultraviolet-crosslinking assays. Co-localization was only detected in a small proportion of RNA foci, suggesting dynamic sequestration rather than irreversible binding. Additional immunohistochemistry demonstrated that neurons with and without RNA foci were equally likely to show nuclear depletion of TDP-43 (χ^2^ *P = *0.75) or poly-GA dipeptide repeat protein inclusions (χ^2^ *P = *0.46). Our findings suggest two non-exclusive pathogenic mechanisms: (i) functional depletion of RNA-processing proteins resulting in disruption of messenger RNA splicing; and (ii) licensing of expanded *C9orf72* pre-messenger RNA for nuclear export by inappropriate association with messenger RNA export adaptor protein(s) leading to cytoplasmic repeat associated non-ATG translation and formation of potentially toxic dipeptide repeat protein.

## Abstract (precis)

Expansion of GGGGCC repeats in *C9orf72* causes familial amyotrophic lateral sclerosis (ALS) and frontotemporal dementia, but the underlying mechanism is unclear. Using RNA pulldown and immunohistochemistry in ALS biosamples, Cooper-Knock *et al*. identify proteins that bind to the repeat expansions. Disrupted RNA splicing and/or nuclear export may underlie *C9orf72*-ALS pathogenesis.

## Introduction

Expanded GGGGCC repeats in intron 1 of the *C9orf72* gene represent the most common cause of familial amyotrophic lateral sclerosis (ALS) and familial frontotemporal degeneration (DeJesus-Hernandez *et al.*, 2011; Renton *et al.*, 2011), though how this genetic change results in neuronal injury is not yet understood. Three potential mechanisms have been proposed: (i) haploinsufficiency through disrupted expression of the expanded allele (DeJesus-Hernandez *et al.*, 2011); (ii) RNA mediated gain-of-function toxicity by the transcribed expanded intronic sequence; and (iii) protein mediated gain-of-function toxicity by dipeptide repeat protein aberrantly translated from the repeat sequence by repeat associated non-ATG translation (Ash *et al.*, 2013; Mori *et al.*, 2013*b*). Evidence for haploinsufficiency is mixed; several groups have reported reduced expression of the *C9orf72* messenger RNA, but this finding is not consistent (Sareen *et al.*, 2013). Furthermore no additional loss of function mutations have been found in the *C9orf72* gene (Harms *et al.*, 2013) and we and others have shown that smaller repeat lengths, which are considered pathogenic (Byrne *et al.*, 2013; Gomez-Tortosa *et al.*, 2013), do not reduce transcription (Cooper-Knock *et al.*, 2013; Xi *et al.*, 2013). More evidence is being gathered for a gain-of-function toxicity mediated either by RNA foci formed from the expanded intron or through repeat associated non-ATG translation.

Recently, a number of studies reported that molecular phenotypes correlated with the presence of RNA foci (Donnelly *et al.*, 2013; Lagier-Tourenne *et al.*, 2013; Lee *et al.*, 2013; Mizielinska *et al.*, 2013; Sareen *et al.*, 2013). Two of these studies corrected the observed phenotype by targeted degradation of the foci using antisense oligonucleotides (Donnelly *et al.*, 2013; Sareen *et al.*, 2013). One study suggested that foci burden in the frontal cortex positively correlated with disease severity in eight patients with *C9orf72* frontotemporal degeneration (Mizielinska *et al.*, 2013). Two of these reports identified co-localization of RNA foci with various proteins (Donnelly *et al.*, 2013; Sareen *et al.*, 2013) and suggested that pathogenic sequestration might be occurring. A similar process has been observed in myotonic dystrophy type 1, another neuromuscular disease caused by an intronic expansion (Jiang *et al.*, 2004). Previously two groups generated candidate binding partners of the GGGGCC repeat expansion, but did not include co-localization studies with RNA foci (Mori *et al.*, 2013*a*; Xu *et al.*, 2013). Further work to characterize protein binding partners of the RNA foci is required, particularly because many of the studies thus far are in disagreement as to the most important interactions.

Observations regarding toxicity of repeat associated non-ATG translation are still at an early stage: the produced dipeptide repeat protein appears to be toxic in a cell model (Zu *et al.*, 2013), but levels of the aberrantly translated protein observed do not correlate with neurodegeneration in autopsy material (Mackenzie *et al.*, 2013). An important question remains over the mechanism by which the transcribed repeat sequence is exported to the cytoplasm to allow repeat associated non-ATG translation. Clearly, normal control of messenger RNA nuclear export would be expected to inhibit this movement. However, several studies report cytoplasmic RNA foci in CNS tissue (Donnelly *et al.*, 2013, Mizielinska *et al.*, 2013).

We have used fluorescence *in situ* hybridization (FISH) to examine the abundance and location of RNA foci in cerebellum, where p62-positive protein inclusion pathology is characteristic of *C9orf72*+ disease (Cooper-Knock *et al.*, 2012), and in motor neurons of the ventral horn. We also examined the relationship between RNA foci and characteristic neuropathology of *C9orf72*+ ALS: first, the loss of nuclear TDP-43 in motor neurons, which is the pathological hallmark of ALS (Neumann *et al.*, 2006) and has been shown to correlate with neuronal loss (Brettschneider *et al.*, 2013); and second, the presence of cytoplasmic aggregates containing dipeptide repeat protein, which are a hallmark of *C9orf72*+ disease (Ash *et al.*, 2013; Mackenzie *et al.*, 2013; Mori *et al.*, 2013b). We have then identified protein binding partners of the RNA repeat expansion, initially in an *in vitro* RNA pulldown assay using both cerebellum and neuronal cell-line extracts, and then subsequently in CNS tissue from *C9orf72*+ patients with ALS by immunohistochemistry. Protein–RNA UV-crosslinking confirmed *in vitro* direct interactions with the repeat sequence. We add novel insights to this growing field and in particular, our focus on motor neurons from the ventral horn of the spinal cord has allowed us to characterize RNA foci and their interactions in the neuronal population most vulnerable to neurodegeneration in ALS.

It should be noted that other groups have observed RNA foci transcribed from the repeat sequence in an antisense direction consisting of a GGCCCC repeat (Gendron *et al.*, 2013; Lagier-Tourenne *et al.*, 2013; Mizielinska *et al.*, 2013); antisense foci were not examined in this study.

## Materials and methods

### Human samples

The study was approved by the South Sheffield Research Ethics Committee and informed consent was obtained for all samples. Brain and spinal cord tissues were donated to the Sheffield Brain Tissue Bank for research with the consent of the next of kin. Immunohistochemistry and RNA FISH were performed on formalin fixed paraffin-embedded tissues from up to five *C9orf72*+ ALS cases, three *C9orf72−* ALS cases and three neurologically normal controls. Lymphoblastoid cells and fibroblasts from three *C9orf72*+ ALS cases, one *C9orf72*+ asymptomatic carrier, three *C9orf72−* ALS cases and three controls were used for RNA FISH. Lymphoblastoid cell lines were obtained from the Wellcome Trust/Motor Neurone Disease Association ALS/MND UK DNA and Lymphoblastoid cell line Bank. Fibroblasts were obtained from the Sheffield MND Biosamples Bank.

### RNA fluorescence *in situ* hybridization

A 5’ TYE-563-labelled LNA (16-mer fluorescent)-incorporated DNA probe was used against the sense RNA hexanucleotide repeat (Exiqon, Inc., batch number 607323). Slides with tissue, lymphoblastoid cells or fibroblasts were fixed in 4% paraformaldehyde for 10 min. Before use, formalin fixed paraffin-embedded tissue sections were deparaffinized. Slides were blocked with hybridization solution [50% formamide, 2× saline sodium citrate (SSC), 100 mg/ml dextran sulphate, 50 mM sodium phosphate pH 7.0] for 3 h at 66°C and then incubated with 400 ng/ml of denatured probe in hybridization solution overnight at 66°C. After hybridization, slides were washed once in 2×SSC/0.1% Tween-20 at room temperature and three times in 0.1× SSC at 65°C. Slides were mounted with mounting medium containing DAPI (Vector Labs, Inc.). All solutions were made with DEPC-treated water.

### Visualization of RNA foci

Primary visualization of foci was performed using a Leica SP5 confocal microscope system with a ×63/1.4 oil immersion objective lens. The presence of foci was assessed within a high resolution (1433 µm^2^ per image, 511 × 511 pixels) *z*-stack made up of images at 0.13-µm intervals through the entire nuclear volume of the cell under consideration.

### Biotinylated RNA pulldown assays

Total extracts were prepared by homogenizing and lysing cells/tissue in RNA-pulldown (RPD) lysis buffer [25 mM Tris pH 7.4, 100 mM NaCl, 1 mM DTT, 10% (v/v) glycerol, 0.5% (v/v) Triton™ X-100]. Lysates were cleared by centrifugation and supernatants taken for experiments. Nuclear extracts from SH-SY5Y cells were prepared using the Dignam method (Dignam *et al.*, 1983). We chose to use two methods of lysis because cell lysis has been shown to influence the composition of ribonucleoprotein complexes (Mili and Steitz, 2004).

AAAAUU_5_ and GGGGCC_5_ RNA molecules with 3’ biotin modifications were used to identify protein binding partners in pulldown assays. 60 μl aliquots of streptavidin sepharose (GE Healthcare) were blocked overnight on a spinning wheel at 4°C with RPD lysis buffer containing 2% bovine serum albumin. Total extracts were lysed in RPD lysis buffer whereas cerebellum homogenates and SH-SY5Y whole cell or nuclear extracts were mixed 1:1 with RPD lysis buffer (2×) supplemented with protease and RNase inhibitors. 1-2 mg of the appropriate total cellular or nuclear lysate was mixed with 15 µg biotin-labelled RNA, incubated at room temperature for 30 min and then on ice for 30 min. Mixtures were then transferred to a 6-cm petri dish and UV irradiated on ice at 0.3 J/cm^2^ in a UV crosslinker (*Fisher*). Mixtures were then applied to blocked streptavidin sepharose and incubated at 4°C for 2 h with agitation. Following binding, beads were washed three times with RPD lysis buffer and then twice with RPD wash buffer (25 mM Tris pH 7.4, 100 mM NaCl, 1 mM DTT). Complexes were eluted by addition of RPD elution buffer (25 mM Tris pH 7.4, 25 mM NaCl, 1 mM EDTA) and 10 µg RNase A followed by agitation at room temperature for 30 min. Eluates were analysed by SDS-PAGE and proteins identified by mass spectrometry or western immunoblotting.

### Mass spectrometry

In solution tryptic digestions were performed on the eluted fractions by the addition of 100 mM final concentration ammonium bicarbonate and 0.1% ProteaseMAX™ surfactant. Trypsin was added to a mass ratio of (1:50) and incubated at 37°C overnight. Digestions were stopped with the addition of 1–2 µl glacial acetic acid and subsequently dried under vacuum. Tryptic digests were resuspended in 0.1% final concentration of trifluoroacetic acid. Five microlitres was used for liquid chromatography–mass spectrometry/mass spectrometry (LC–MS/MS) analysis. Peptides were separated using an UltiMate™ 3000 RSLC nano liquid chromatography system (Dionex), using a 150 mm × 75 µm I.D. PepMap™ reversed phase column (Dionex). Linear gradient elution was performed from 95% buffer A (0.1% formic acid) to 50 % buffer B (0.1% formic acid, 95 % acetonitrile) at a flow rate of 300 nl/min in 60 mins. MS/MS analysis was performed using a maXis UHR TOF mass spectrometer (Bruker Daltonics) using an automated acquisition approach. MS and MS/MS scans (m/z 50–2000) were acquired in positive ion mode. Lock mass calibration was performed using HP 1221.990364. Line spectra data were then processed into peak list by data analysis using the following settings. The sum peak finder algorithm was used for peak detection using a signal to noise ratio of 10, a relative to base peak intensity of 0.1% and an absolute intensity threshold of 100. Spectra were deconvoluted and the peak lists exported as Mascot Generic Files (MGF) and searched using Mascot 2.2 server (Matrix Science). The Swiss-Prot database (Swiss-Prot Release 10.5m5, 20 April 2010, 516604 sequences) was searched using the following parameters (analysis peptide tolerance = ±0.01 Da, MS/MS tolerance = ±0.01 Da and peptide charge 2+ and 3+). Search parameters were as follows: enzyme; trypsin; fixed modifications: carbamidomethyl (C); variable modifications: deamidation (NQ), oxidation (M); maximum missed cleavages: 1. Deamidation (NQ) were chosen as variable modifications. Additionally, we also used a peptide MOWSE score of <25 as a cut-off as calculated by Mascot. The false discovery rate was estimated to be 1% for peptide IDs after searching reverse databases. Protein identifications were based on a minimum of two unique peptides.

### RNA-binding ultraviolet crosslinking assays

RNA-binding assays were carried out as described previously (Hautbergue *et al.*, 2008, 2009). GGGGCC_5_ RNA was 5’ end labelled with γ^32^P-ATP using T4 polynucleotide kinase (Fermentas). Reaction mixes were made up in RNA binding buffer [15 mM HEPES pH 7.5, 150 mM NaCl, 5 mM MgCl_2_, 10% (v/v) glycerol, 0.05% (v/v) Tween-20] with 50 ng radiolabelled RNA and 5 µg purified recombinant protein. Mixes were incubated for 20 min at room temperature and 20 min on ice before being UV-irradiated on ice at full power. Complexes were analysed by SDS-PAGE and stained with Coomassie blue before being vacuum-dried and exposed on a phosphoimage screen.

### Immunohistochemistry

The following antibodies were used for immunohistochemistry: anti-TDP-43 (Proteintech 10782-2-AP) anti-FUS (Novus NB100-2599), anti-hnRNP H1/F (Abcam ab10689), anti-hnRNP A1 (Abcam ab5832, 9H10 clone), anti-hnRNP D (Proteintech 12770-1-AP), anti-SRSF1 (phosphor, Abcam ab11826), anti-SRSF2 (Abcam ab30817), anti-ALYREF (Sigma, clone 11G5) and anti-hnRNP C1/C2 (Abcam ab10294). Poly-(Gly-Ala) dipeptide repeat protein was detected using anti-GA antibodies (mouse, clone 5F2) as previously described (Mackenzie *et al.*, 2013). Antigen retrieval was performed by 10–30-min microwave in EDTA at pH 8.0 for all antibodies except anti-SRSF1, anti-ALYREF and anti-TDP-43 where antigen retrieval involved microwave 10–20 min in trisodium citrate at pH 6.5, and for anti-hnRNP H/F where no specific antigen retrieval was performed. After incubation with the primary antibodies, slides were washed in PBS and incubated in species specific Alexa Fluor® 488-conjugated secondary antibodies.

## Results

### RNA fluorescence *in situ* hybridization

**Figure 1:**
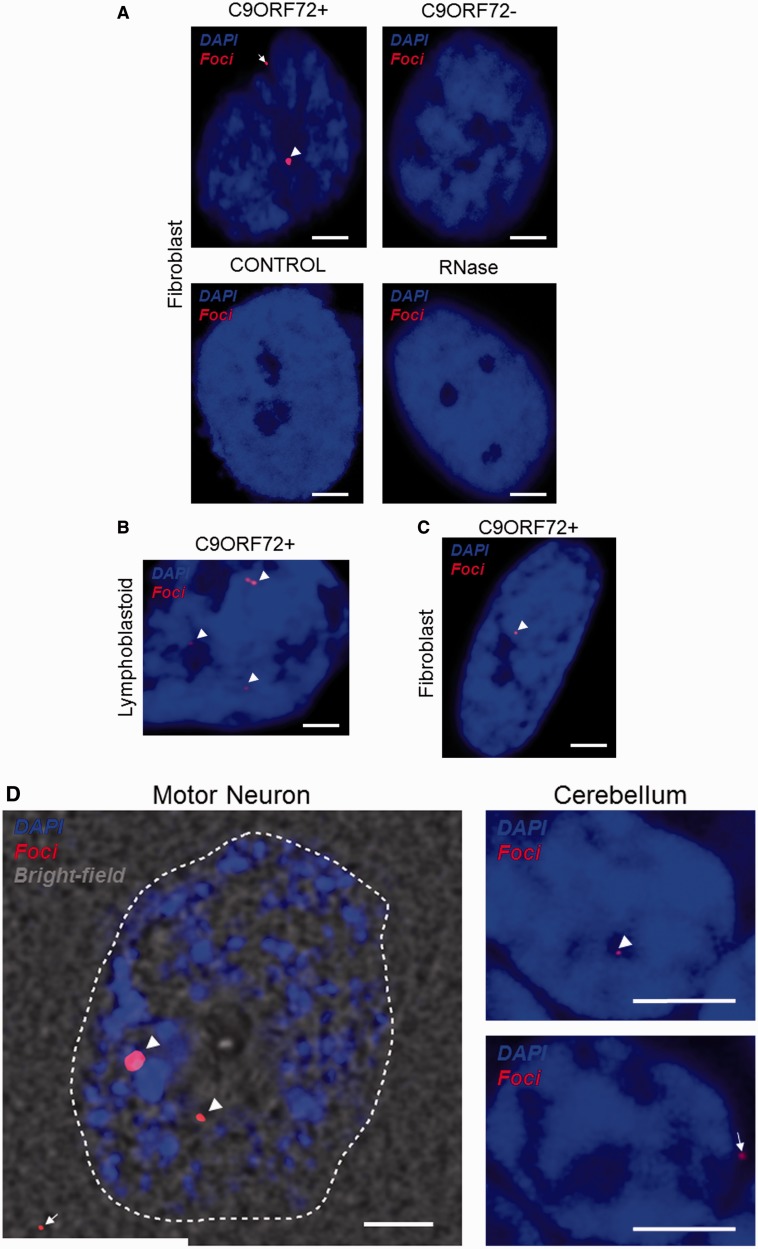

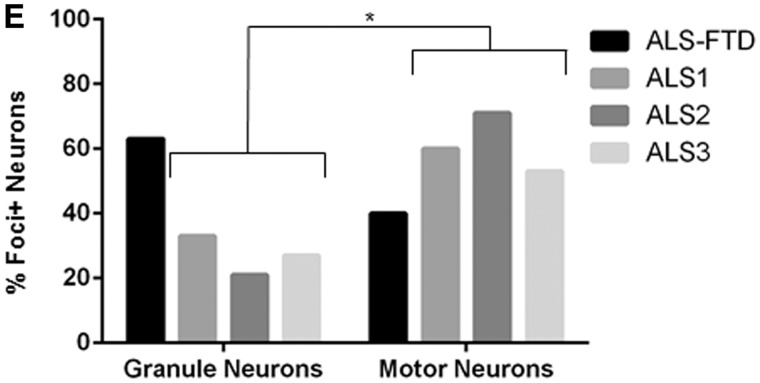
RNA FISH shows GGGGCC expanded RNA foci are found in peripheral cells and CNS tissue from *C9orf72*+ patients but not from *C9orf72−* ALS cases or controls. RNA foci (arrowheads) are present in fibroblasts (**A**), lymphoblastoid cells (**B**) and CNS tissue (**D**) from *C9orf72*+ patients with ALS, and in fibroblasts from a *C9orf72*+ asymptomatic carrier (**C**). RNA foci are ablated by RNase treatment (**A**). RNA foci are predominantly nuclear but cytoplasmic foci are observed in peripheral cells and CNS tissue (**A** and **D**, arrows). Abundance of foci in cerebellar granule cells and motor neurons has been quantified (**E**), in those cases where the initial clinical presentation was ALS the proportion of foci+ motor neurons is significantly higher (*P < 0.05). Scale bar = 3 µm. FTD = frontotemporal degeneration.

The presence of RNA foci clearly distinguished fibroblasts, lymphoblastoid cells and CNS tissue from *C9orf72*+ patients with ALS compared to *C9orf72−* patients with ALS and neurologically normal control subjects (Fig. 1A–D). To validate our RNA FISH methodology, discrete nuclear foci-like staining was quantified in a blinded study of 50 cerebellar granule neurons from each of nine cases: three *C9orf72*+ patients with ALS, three *C9orf72−* patients with ALS and three control subjects. In *C9orf72*+ tissue the average proportion of neurons containing nuclear RNA foci was 39% (range 21–63%); in three *C9orf72−* cases with ALS the average proportion of neurons containing foci-like staining was 1.6% (range 1.1–2.5%); in normal controls the average proportion of neurons containing foci-like staining was 1.4% (range 1.3–1.6%). Only seven foci-like objects were observed in 300 neurons from the six non-*C9orf72*+ cases and never was more than one focus-like object was observed in a single cell; in contrast the average rate in *C9orf72*+ tissue was two foci per cell. RNase treatment in fibroblasts ablated foci, illustrating that the labelled product is RNA and in agreement with previous studies (Fig. 1A).

It is noteworthy that RNA foci were identified in fibroblasts derived from an asymptomatic *C9orf72*+ carrier (Fig. 1C). In four *C9orf72*+ cases the proportion of foci+ cerebellar granule neurons was quantified and compared to the proportion of foci+ motor neurons in the ventral horn (Fig. 1E). More than 35 cells of each neuron-type were examined in each case. Three of the cases presented initially with ALS (Supplementary Fig. 1); in these patients the average proportion of foci+ neurons was significantly higher in the ventral horn (61% versus 27%, *t-*test *P < *0.05). In the fourth case, who presented with frontotemporal degeneration and later developed ALS, the pattern was reversed (40% versus 63%). Foci were primarily nuclear, however, some cytoplasmic foci were also observed in fibroblasts, cerebellar granule cells and in motor neurons (Fig. 1C).

### Identification of binding partners of the *C9orf72* repeat expansion

**Figure 2:**
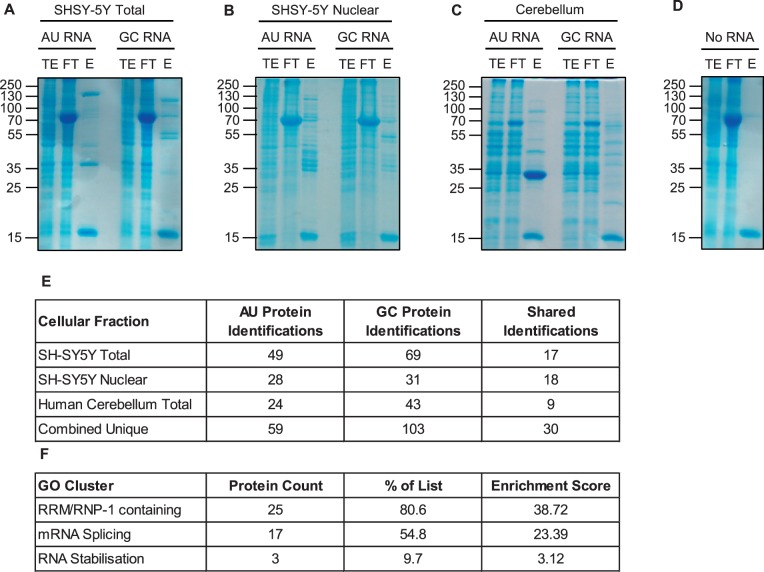
5’-[AAAAUU]_5-_ and 5’-[GGGGCC]_5_ RNAs sequester distinct sets of proteins from human neuronal cell line fractions and dissected human cerebellar tissue. Pulldown assays using biotinylated RNAs (no RNA, 5’-[AAAAUU]_5-_ or 5’-[GGGGCC]_5_) and extracts from total or nuclear fractions of SH-SY5Y cells, or human cerebellar tissue; I = input (1%); FT = flow through (1%); E = eluted (25%) (**A–D**). Mass spectrometry (MS) analysis of proteins co-purified with biotinylated RNAs (**E**). Gene ontology (GO) enrichment of SH-SY5Y nuclear hits (**F**).

We generated 3’ biotinylated RNAs with the following sequences: 5’-[AAAAUU]_5_-Bio-3’ and 5’-[GGGGCC]_5_-Bio-3’. It has been demonstrated that the GGGGCC repeat expansion can form RNA G-quadruplexes *in vitro*, with the smallest repeating unit consisting of four repeats (Fratta *et al.*, 2012; Reddy *et al.*, 2013). To identify proteins interacting with the biotinylated RNAs, RNAs were preincubated with protein extracts and resulting complexes fixed by UV-irradiation. The RNA bait and bound proteins were captured using streptavidin sepharose and eluted after RNase A digestion. We used whole cell lysates of the human neuronal cell line SH-SY5Y, SH-SY5Y nuclear extract and dissected human cerebellum whole extract (Fig. 2A–C). Controls without RNA bait were processed in parallel (Fig. 2D). Eluted proteins were identified by mass spectrometry. In total, 103 unique proteins were identified that bind GGGGCC_5_, the majority of which did not bind to AAAAUU_5_ (Fig. 2E and Supplementary material).

Gene ontology (GO) enrichment analysis of each GGGGCC_5_-derived list of bound proteins was carried out using the Database for Annotation, Visualization and Integrated Discovery (DAVID) (Huang da *et al.*, 2009*a*, *b*). This yielded functional categories associated with aspects of messenger RNA metabolism including splicing and stabilization, and an RNA recognition motif-containing class (Supplementary Fig. 2). This was particularly striking in the list of GGGGCC RNA-binders isolated from nuclear extracts of the SH-SY5Y human neuronal cell line (Fig. 2F). Another strongly represented group was messenger RNA export adaptors, which promote nuclear export via remodelling of the NXF1/TAP export receptor (Hautbergue *et al.*, 2008), including ALYREF and the shuttling splicing factors SRSF1 (SF2/ASF), SFRS3 (SRp20) and SFRS7 (9G8) (Walsh *et al.*, 2010).

### Cellular distribution of RNA foci and RNA recognition motif-containing proteins

**Figure 3:**
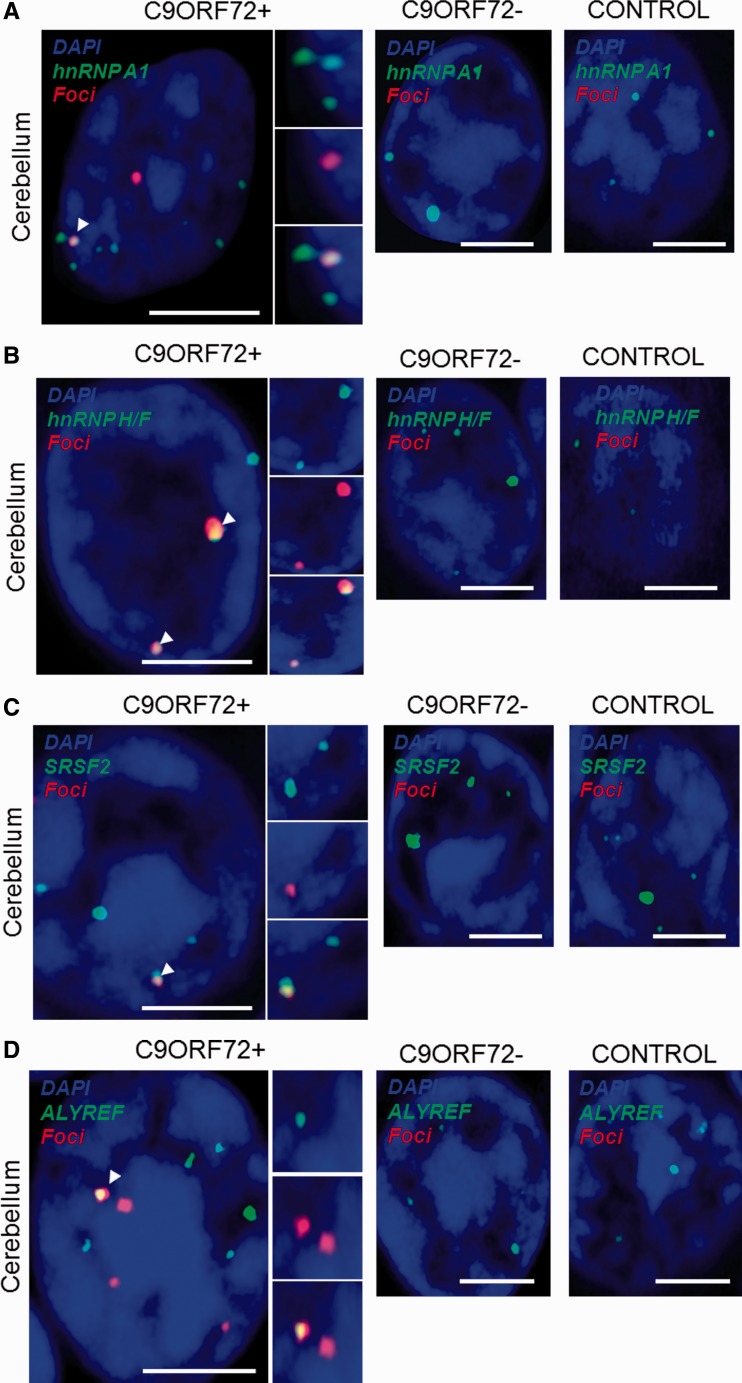

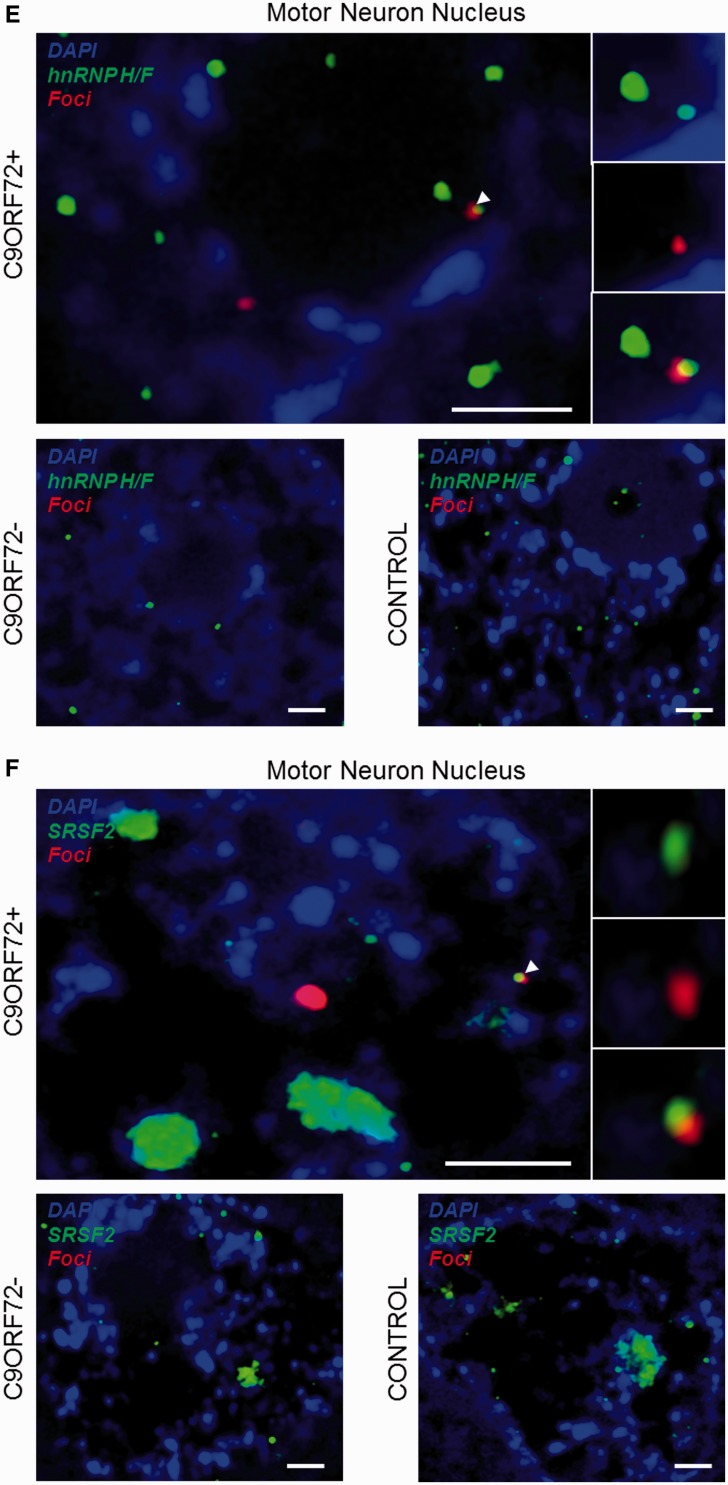

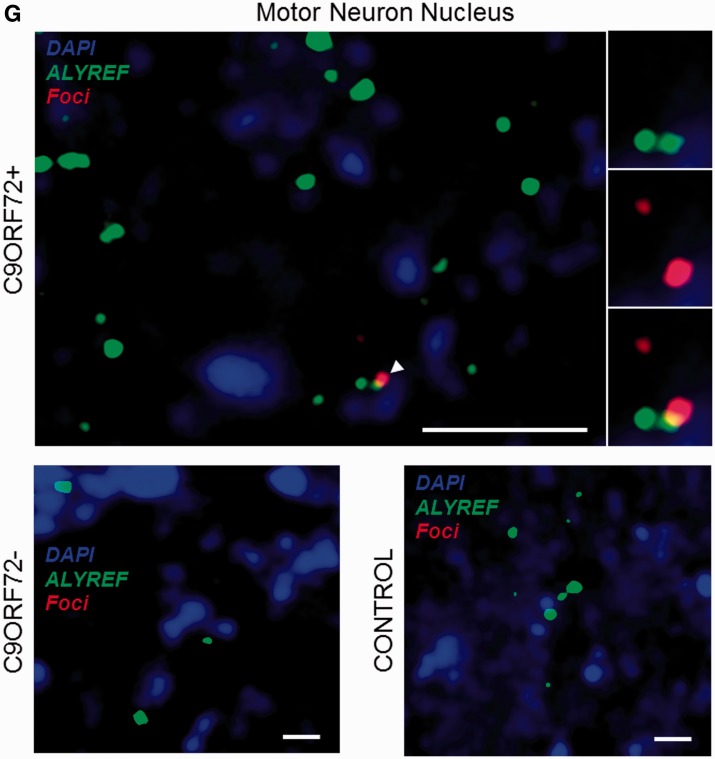
Combined RNA FISH and immunohistochemistry demonstrate co-localization of nuclear speckle components with RNA foci in CNS tissue. hnRNP A1 (**A**), hnRNP H1/F (**B**), SRSF2 (**C**) and ALYREF (**D**) are observed to co-localize with RNA foci (arrowheads) in cerebellar granule cells from *C9orf72*+ patients with ALS. hnRNP H1/F (**E**), SRSF2 (**F**) and ALYREF (**G**) are observed to co-localize with RNA foci (arrowheads) in nuclei of motor neurons from *C9orf72*+ patients with ALS. Co-localization events are enlarged and unmerged protein and RNA foci are shown for comparison. The normal staining pattern of the two proteins in *C9orf72−* cases with ALS and control subjects is included for comparison. Scale bar = 3 µm.

We used confocal microscopy to validate *in vivo* some of the hits identified by mass spectrometry. For this purpose, eight well-described RNA recognition motif-containing proteins including splicing factors and messenger RNA nuclear export adaptors were prioritized and selected depending on available and efficacious commercial antibodies. The distribution of each protein relative to RNA foci was examined in approximately 200 cerebellar granule neurons and 50 motor neurons from a minimum of three *C9orf72*+ cases with ALS. Simultaneous co-staining was carried out in parallel in *C9orf72−* cases with ALS and neurologically normal control subjects. For all tested candidates, overall cellular protein distribution was not grossly different between *C9orf72*+ cases, *C9orf72−* cases and controls except for areas where co-localization was demonstrated. In cerebellar granule cells we demonstrated co-localization of hnRNP A1, hnRNP H1/F, ALYREF and SRSF2 with 27%, 30%, 26% and 33% of RNA foci, respectively (Fig. 3A–D). In motor neurons, the cell type most vulnerable to the neurodegenerative process in ALS, we demonstrated co-localization of hnRNP H1/F, ALYREF and SRSF2 with 19%, 29% and 30% RNA foci, respectively (Fig. 3E–G). In contrast, we were unable to detect any evidence of co-localization of other identified GGGGCC-binding partners SRSF1, FUS, hnRNP C or hnRNP D with sense foci in either the cerebellar granule layer or the ventral horn (Supplementary Fig. 3).

**Figure 4:**
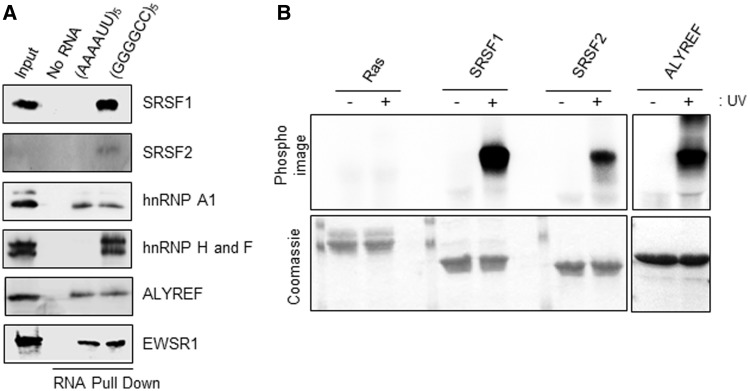
Identified RNA-binding candidates interact specifically and directly with GGGGCC_5_ RNA. (**A**) Neuronal SH-SY5Y whole cell extract was incubated with either no RNA, AU-rich or GC-rich biotinylated RNA coated onto streptavidin beads before UV-cross linking. Bound proteins were eluted using RNase A and further identified using SDS-PAGE and western immunoblotting with the indicated antibodies. It is noted that the weak signal for SRSF2 is due to difficulty finding an antibody that is efficacious in western immunoblotting. The anti-hnRNP H1/F antibody recognizes both proteins, which are similar (Garneau *et al.*, 2005). (**B**) Hexa-histidine-tagged recombinant SRSF1 11-196, GB1-tagged SRSF2 9-101 and ALYREF full length were expressed in *E. coli* and purified using metal ion affinity chromatography in 1 M NaCl containing buffers to remove potentially bound RNA from *E. coli* (*bottom*). GGGGCC_5_ RNA was separately end-labelled with poly nucleotide kinase using [γ-^32^P]-ATP, before incubation with purified proteins. RNA was covalently bound (+) or not (−) after UV irradiation. Absence of radioactive signal (*top*; PhosphoImage) in absence of UV irradiation demonstrates specificity of direct binding observed after UV treatment. All gels shown in the different panels were exposed simultaneously for the same amount of time (5 h).

For six of the proteins identified in the mass spectrometry analysis, including those proteins observed to co-localize with RNA foci *in vivo*, specificity of interaction with the (GGGGCC)_5_ RNA was assessed using RNA pull down assays from whole neuronal SH-SY5Y cell extract and western immunoblotting (Fig. 4A). Direct binding of some of these proteins to (GGGGCC)_5_ RNA repeat was also confirmed in a UV-cross linking assay using radiolabelled RNA and recombinant proteins which were expressed and purified from *E. coli* (Fig. 4B).

We also examined the co-incidence of RNA foci with depletion of TDP-43 from the nuclei of motor neurons of *C9orf72*+ patients with ALS. Mislocalization of TDP-43 is the pathophysiological hallmark of ALS (Neumann *et al.*, 2006). All surviving motor neurons were examined in formalin fixed paraffin-embedded sections from three *C9orf72*+ ALS cases. The majority of cells with nuclear depletion of TDP-43 contained nuclear RNA foci, but this was not significantly different to the proportion of cells with nuclear TDP-43 expression that contained RNA foci (66% versus 60%, χ^2^ *P = *0.75) (Supplementary Fig. 4).

In view of our prediction that the repeat sequence might sequester several proteins important for messenger RNA export, we wanted to explore the relationship between repeat associated non-ATG translated protein and RNA foci in specific neuronal populations. As expression of dipeptide repeat proteins is reported to be rare in the ventral horn of *C9orf72*+ patients with ALS (Mackenzie *et al.*, 2013), we chose to focus on cerebellar granule cells. Fifty per cent of the neurons which stained for poly-GA, the most abundant dipeptide repeat protein, contained nuclear RNA foci; this was not significantly different to the proportion of neurons with nuclear RNA foci which did not stain for poly-GA (50% versus 40%, χ^2^ *P = *0.46) (Supplementary Fig. 5).

## Discussion

There is an urgent need to understand the mechanisms of neuronal injury in *C9orf72*+ disease. This genetic variant is the most common identified cause of ALS and frontotemporal degeneration. We and others (DeJesus-Hernandez *et al.*, 2011; Donnelly *et al.*, 2013; Lagier-Tourenne *et al.*, 2013; Lee *et al.*, 2013; Mizielinska *et al.*, 2013; Sareen *et al.*, 2013) have identified RNA foci formed from the intronic GGGGCC repeat sequence in peripheral cells and CNS tissue from *C9orf72*+ patients. We have particularly focused on characterizing RNA foci within spinal motor neurons, which are the primary target of pathology in ALS. Indeed we have shown that RNA foci are present in a higher proportion of motor neurons of the ventral horn compared to cerebellar granule cells in patients where the initial clinical presentation was ALS; in a single patient where the initial clinical presentation was with extra-motor disease the opposite was true. This is consistent with toxicity initiated by RNA foci. However, this finding will require validation in a larger number of cases.

We have identified a number of putative binding partners of the RNA repeat expansion which are consistent with previous observations (Lee *et al.*, 2013; Mori *et al.*, 2013*a*; Sareen *et al.*, 2013; Xu *et al.*, 2013). Of the RNA recognition motif-containing proteins we found to be co-localized with RNA foci in *C9orf72*+ tissue, hnRNP A1 (Sareen *et al.*, 2013), hnRNP H1/F and SRSF2 (Lee *et al.*, 2013) have been similarly observed by others. Interestingly, our study provides the first evidence for co-localization of RNA foci with the general messenger RNA nuclear export adaptor ALYREF (Stutz *et al.*, 2000). Observed co-localization with RNA recognition motif-containing proteins was present in a relatively low percentage of RNA foci. We suggest that this is consistent with a process of dynamic sequestration. Indeed, irreversible binding of these candidates, many of which are key regulators of essential processes such as pre-messenger RNA splicing, is unlikely to be consistent with the relatively late age of disease onset seen in *C9orf72*+ patients. The key pathogenic step may be downstream from protein sequestration by the expansion, such as export of the repeat expansion to enable repeat associated non-ATG translation or an accumulation of aberrant splicing events. Importantly we have confirmed a direct interaction *in vitro* between our protein candidates and the GGGGCC repeat RNA by UV-crosslinking.

SRSF2 is a well-known marker for nuclear speckles, nuclear domains implicated in the storage and supply of splicing factors to active transcription sites (Spector and Lamond , 2011). All of the proteins we have shown to co-localize with RNA foci, many of the binding partners identified in our RNA pulldown, and a number of the proteins implicated in genetic variants of ALS including TARDBP, EWSR1, FUS, HNRNPA1 and HNRNPA2B1, have been localized to nuclear speckles (Zhou *et al.*, 2000; Saitoh *et al.*, 2004; Casafont *et al.*, 2009). Other neuromuscular diseases have been associated with depletion of normal components of nuclear speckles including myotonic dystrophy type 1 (Smith *et al.*, 2007; Bengoechea *et al.*, 2012). It is possible that disruption of the normal function of nuclear speckles, either by a direct mutation of one of the key protein components, or via RNA foci-mediated dynamic depletion of essential protein constituents, is a key pathogenic mechanism in ALS. Analysis of the transcriptome of pathologically affected neurons will be key to elucidating whether the interactions we have identified have a toxic effect through disruption of messenger RNA splicing.

We provide evidence for cytoplasmic RNA foci, not only in peripheral cells and in the cerebellar granule layer, but also in motor neurons from the ventral horn of the spinal cord. Cytoplasmic localization of RNA foci formed from an intronic repeat sequence in peripheral cells might be consistent with extrusion during mitosis. However, in non-dividing neurons of the cerebellum and ventral horn this is not a possibility. The alternative scenario relates to nuclear export of the transcribed GGGGCC repeat expansion. Our RNA pulldown screen for binding of the repeat expansion identified multiple messenger RNA export adapters including ALYREF (Stutz *et al.*, 2000), SRSF1, SFRS3 and SFRS7 (Huang *et al.*, 2003; Hargous *et al.*, 2006; Tintaru *et al.*, 2007). In the case of ALYREF we have also demonstrated co-localization with RNA foci by immunohistochemistry, and a direct interaction with the expansion by protein-RNA UV-crosslinking. An interesting possibility is that local enrichment of messenger RNA export adaptors onto *C9orf72* GGGGCC repeat pre-messenger RNA molecules overrides the normal nuclear retention of pre-messenger RNA, for example through an inappropriate interaction of ALYREF with the TAP/NXF1 nuclear export receptor. It seems unlikely that the RNA foci are exported intact, particularly because of their size and activity of DEAD box RNA helicases such as Dbp5/DDX19, on the cytoplasmic side of the nuclear pore which would be expected to unwind G-quadruplex structures (Linder, 2008). However, it is conceivable that aberrantly expanded *C9orf72* pre-messenger RNA molecules are exported from the nucleus and reform into foci within the cytoplasm.

Nuclear export of GGGGCC repeat RNA is likely to be a key step leading to repeat associated non-ATG translation in the cytoplasm. If dipeptide repeat proteins formed in this manner are eventually identified as the key mediator of pathogenicity in *C9orf72*+ disease then blocking this export represents an attractive therapeutic target. One report has suggested that the production of repeat associated non-ATG translated protein is mutually exclusive to the presence of RNA foci (Donnelly *et al.*, 2013). In contrast, we found an equal proportion of poly-GA staining in neurons that did or did not contain RNA foci.

We did not observe a significant correlation between nuclear loss of TDP-43 and the presence of RNA foci. This does not mean that RNA foci are not instrumental in the disease pathogenesis, but may reflect the fact that they occur significantly upstream of TDP-43 mislocalization. In this regard it is important to note that we and others (Lagier-Tourenne *et al.*, 2013) have identified RNA foci in fibroblasts derived from asymptomatic *C9orf72*+ carriers.

We await confirmation of our findings by other groups. We have suggested two ways in which the interactions identified may be pathogenic: (i) through disruption of the normal function of factors involved in nuclear speckles and thus messenger RNA splicing; and (ii) through inappropriate licensing of the transcribed *C9orf72* expansion for nuclear export thereby facilitating repeat associated non-ATG translation. Either or both may be important, but it should be noted that if inappropriate licensing of RNA foci for export is a key pathogenic step, then overexpression of the sequestered protein will not be of therapeutic benefit and may even have an adverse effect. On the contrary if loss of the normal function of these proteins is most important, then increasing the nuclear expression of proteins sequestered by the expansion may be of value as a neuroprotective strategy.

## Supplementary Material

Supplementary Data

## Abbreviations

ALS: amyotrophic lateral sclerosis
FISH: fluorescence *in situ* hybridization
