# Supplementary material for: Sequestration of multiple RNA recognition motif-containing proteins by C9orf72 repeat expansions
Source: Brain. 2014 May 27;137(7):2040–51. doi: 10.1093/brain/awu120 (PMC4065024; doi:10.1093/brain/awu120)
Supplement: Supplementary Data [file supp_137_7_2040__index.html]

Sequestration of multiple RNA recognition motif-containing proteins by C9orf72 repeat expansions — Supplementary Data 

# Sequestration of multiple RNA recognition motif-containing proteins by *C9orf72* repeat expansions

## Supplementary Data

files

**Files in this Data Supplement:**

- Supplementary Data - xls file
